# Ameliorative Effect of Mannuronate Oligosaccharides on Hyperuricemic Mice via Promoting Uric Acid Excretion and Modulating Gut Microbiota

**DOI:** 10.3390/nu15020417

**Published:** 2023-01-13

**Authors:** Biqian Wei, Pengfei Ren, Ruzhen Yang, Yuan Gao, Qingjuan Tang, Changhu Xue, Yuming Wang

**Affiliations:** 1 College of Food Science and Engineering, Ocean University of China, Qingdao 266100, China; 2 Laboratory for Marine Drugs and Bioproducts, Qingdao National Laboratory for Marine Science and Technology, Qingdao 266100, China

**Keywords:** hyperuricemia, mannuronate oligosaccharides, uric acid excretion, gut microbiota

## Abstract

Mannuronate oligosaccharide (MOS) is α-D-mannuronic acid polymer with 1,4-glycosidic linkages that possesses beneficial biological properties. The aim of this study was to investigate the hypouricemic effect of MOS in hyperuricemic mice and demonstrate the possible protective mechanisms involved. In this research, 200 mg/kg/day of MOS was orally administered to hyperuricemic mice for four weeks. The results showed that the MOS treatment significantly reduced the serum uric acid (SUA) level from 176.4 ± 7.9 μmol/L to 135.7 ± 10.9 μmol/L (*p* < 0.05). MOS alleviated the inflammatory response in the kidney. Moreover, MOS promoted uric acid excretion by regulating the protein levels of renal GLUT9, URAT1 and intestinal GLUT9, ABCG2. MOS modulated the gut microbiota in hyperuricemic mice and decreased the levels of *Tyzzerella*. In addition, research using antibiotic-induced pseudo-sterile mice demonstrated that the gut microbiota played a crucial role in reducing elevated serum uric acid of MOS in mice. In conclusion, MOS may be a potential candidate for alleviating HUA symptoms and regulating gut microbiota.

## 1. Introduction

Hyperuricemia (HUA) is a metabolic disease caused by disorders of purine metabolism or disturbance of uric acid (UA) excretion [1]. With the alteration of lifestyle and diet structure, the prevalence of hyperuricemia is increasing yearly across the globe, and it is even up to 17.4% in China [2,3,4]. Hyperuricemia is a risk factor for cardiovascular illness, kidney disease, and metabolic syndrome [5].

Uric acid is the final product of purine metabolism. Purines are transformed to UA in the liver by xanthine oxidase (XOD). The kidney and gut are the two primary excretory organs for uric acid. Approximately two-thirds of the body’s uric acid is filtered out by the kidney and eliminated in the urine, while the remaining one-third is ejected through the gut [6]. Uric acid excretion is determined by the equilibrium between uric acid reabsorption and secretion, in which uric acid transporters play a significant role [7]. Urate transporter 1 (URAT1), glucose transporter 9 (GLUT9), and the sodium-coupled monocarboxylate transporter (SMCT) are mostly responsible for uric acid reabsorption, whereas ATP-binding cassette superfamily G (White) member 2 (ABCG2), multidrug resistance associated protein 4 (MRP4), and the organic anion transporters (OAT1, OAT2, and OAT3) are responsible for uric acid secretion [8,9,10]. A disorder of uric acid excretion due to aberrant expression of urate reabsorption or secretion transporters can lead to hyperuricemia. The therapeutically utilized uric acid-lowering medications, XOD inhibitors (allopurinol and febuxostat, etc.), and uricosuric agents (benzbromarone, etc.) show great effectiveness but are associated with harmful side effects, including impairment of liver function or injury [11,12,13]. Some natural products, including chicory, green alga Enteromorpha prolifera polysaccharide, and fucoidan, have been described in the literature to have excellent anti-hyperuricemia effects with hardly any toxicity [14,15,16]. In recent years, the finding of natural compounds promising a decrease in serum uric acid levels has been a research priority.

Kelp and *Undaria pinnatifida* are among the most widely consumed brown seaweeds in the world [17]. Extracts of brown algae are researched for their neuroprotective, anti-inflammatory, antioxidant, and anti-tumor properties [18]. Algin is the prominent active ingredient in brown algae extract, accounting for between 10 to 40 percent of the dry weight of brown algae. It is utilized extensively in the food and medical industries, with an annual global production of around 30,000 tons [19]. Algin can be further degraded biologically or chemically, producing alginate oligosaccharides with various structures, the most physiologically relevant of which is mannuronate oligosaccharide (MOS), a -D-mannuronic acid polymer connected by 1,4-glycosidic linkages [20]. MOS is researched and proven to have biological activities such as anti-inflammatory [21], anti-insulin resistance [22], obesity inhibition [19], and neuroprotective effects [23]. It has tremendous potential in the creation of innovative functional foods and drugs.

Uric acid is a strong pro-inflammatory agent, thus persistent HUA promotes NLRP3 inflammasome activation and inflammatory cytokine secretion, leading to a renal inflammatory phenomenon and, hence, further aggravating HUA [24]. Being an anti-inflammatory active ingredient, MOS may exert a potentially alleviating effect on hyperuricemia. Studies have shown that intestinal flora correlates intimately with the development of HUA [25]. The intestinal flora can not only secrete active enzymes (e.g., XOD enzymes) participating in purine metabolism [26], but also regulate the expression of uric acid transporters through metabolites such as acetate, propionate, and butyrate, thereby influencing intestinal urate excretion [27]. Differences in the gut microbiota composition between HUA patients and normal individuals have been demonstrated, and gut microbiota imbalance can cause disorders of UA excretion and activation of inflammation-related signaling pathways, resulting in more severe HUA [28,29]. MOS was reported to act as a prebiotic to regulate gut microbiota and enhance short-chain fatty acids (SCFAs) production [30]. Thus, in this study, we will investigate whether MOS can ameliorate hyperuricemia via modulating gut microbiota.

This study aimed to investigate the ameliorative effects and potential mechanisms of mannuronate oligosaccharides (MOS) in hyperuricemic mice. We analyzed the implication of MOS on uric acid excretion by gene and protein expression of kidney and intestinal uric acid transporters. The variations of intestinal flora were determined by SCFAs content measurement and 16S rRNA sequencing. Lastly, the crucial role of gut microbiota in the mechanism of MOS in alleviating hyperuricemia was explored deeply through antibiotic-treated mice.

## 2. Materials and Methods

### 2.1. Materials and Reagents

Mannuronate oligosaccharides (MOS) were purchased from Qingdao BZ Oligo Biotech Co., Ltd. (Qingdao, China). Potassium oxonate (PO), allopurinol, 0.5% Sodium carboxymethyl cellulose (CMC-Na) solution, vancomycin, ampicillin, metronidazole, neomycin, 2-ethylbutyric acid, acetic acid (Ace), propionic acid (Pro), butyric acid (But), isobutyric acid (Isobut), valeric acid (Val) and isovaleric acid (Isoval) were purchased from Shanghai yuanye Bio-Technology Co., Ltd. (Shanghai, China). Animal feeds were purchased from Trophic Animal Feed High-Tech Co., Ltd. (Nantong, China). Serum uric acid (UA), blood urea nitrogen (BUN), adenosine deaminase (ADA), and xanthine oxidase (XOD) assay kits were purchased from Nanjing Jiancheng Bioengineering Institute (Nanjing, China). Elisa kits for interleukin-1β (IL-1β), interleukin-12 (IL-12) and interleukin-18 (IL-18) were purchased from Suzhou Calvin Co., Ltd. (Suzhou, China). The primers XOD, ADA, GLUT9, and URAT1 were purchased from Sangon Biotech (Shanghai) Co., Ltd. (Shanghai, China). Protein antibodies URAT1, GLUT9, and ABCG2 were purchased from ABclonal Technology Co., Ltd. (Wuhan, China). All other chemicals and reagents used were of analytical quality.

### 2.2. Animals

Six- to eight-week-old healthy male Balb/c mice (18–20 g) were provided by Vital River Laboratories (Beijing, China) and kept in a 12 h day/night cycle under conditions of 22–25 °C, 40–60% relative humidity. Mice were given a standard laboratory pelleted diet (AIN93G) and water ad libitum for 1 week for acclimatization until they reached the desired weight (22 g/mouse) for the experiments. All experiments were performed in accordance with the National Institutes of Health guide for the care and use of Laboratory animals (NIH Publications No. 8023, revised 1978), and the animal experimental protocol was reviewed and approved by the Animal Experiment Ethics Committee of Ocean University of China (ethical approval code: SPXY2021080501 and SPXY2022051902).

### 2.3. Experimental Designs

The first set of experiments was designed to investigate the role of MOS in ameliorating hyperuricemia in mice and the possible molecular mechanisms. After 7 days of acclimatization feeding, the animals were randomly divided into a normal control group (Normal, *n* = 11) and a modeling group (*n* = 33). The modeling group consumed a high-yeast diet (25%) and was injected intraperitoneally with a 0.5% Sodium carboxymethyl cellulose (CMC-Na) solution containing potassium oxyzincate (200 mg/kg/2d) once every two days at 9:00 a.m. for four weeks. The Normal group, similarly, consumed a standard laboratory pelleted diet (AIN93G) and received an equivalent volume of 0.5% CMC-Na intraperitoneally every two days. After modeling for hyperuricemia for four weeks, the mice were determined to be hyperuricemic by measuring the level of serum UA, which was found to be significantly higher in the modeling group than in the Normal group. Then, the modeling group was randomly divided into (i) the hyperuricemic model group (Model), (ii) the positive drug allopurinol group (Allopurinol), and (iii) the mannuronate oligosaccharides group (MOS) (*n* = 11 per group). Group (i) functioned as a control and had unrestricted access to water. Group (ii) acted as positive controls and received allopurinol (10 mg/kg/day) orally for 4 consecutive weeks. Group (iii) received MOS (200 mg/kg/day) through oral administration for 4 consecutive weeks.

**Figure 1:**
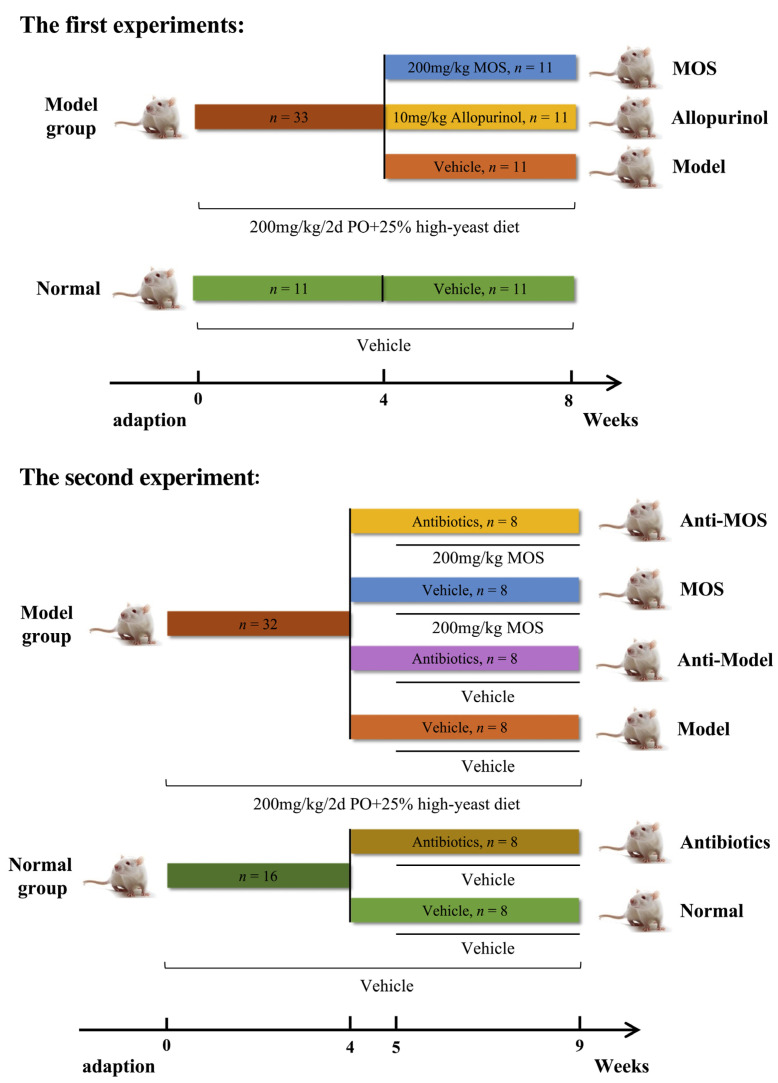
Experimental designs.

The second experiment was designed to examine the effect of gut microbiota in MOS in lowering uric acid levels in mice with hyperuricemia. After the adaptation feeding for 7 days, the animals were randomly divided into the normal group (*n* = 16) and the modeling group (*n* = 32). The modeling group was fed a high-yeast diet (25%) and a 0.5% CMC-Na solution containing potassium oxyzincate (200 mg/kg/2d) was injected intraperitoneally once every 2 days starting at 9 a.m. for 4 weeks to induce hyperuricemia. Then, a mixture of vancomycin (1.25 mg/mL), ampicillin (2.5 mg/mL), neomycin (2.5 mg/mL), and metronidazole (2.5 mg/mL) was added to sterile drinking water for 7 consecutive days to establish a pseudo-sterile mouse model [27,31]. After successful modeling, the normal group was randomly divided into 2 groups (8 animals each): normal control group (Normal group), normal + antibiotics group (Antibiotics); and the modeling group was randomly divided into 4 groups (8 animals each): hyperuricemia model control group (Model group), hyperuricemia + antibiotics group (Anti-Model), hyperuricemia + MOS group (MOS group), and hyperuricemia + MOS + antibiotics group (Anti-MOS). The treatment therapy was strictly in line with the first experiment. To preserve relative sterility in the intestines of the mice during therapy, the same antibiotic cocktail was provided orally every three days in the groups Antibiotics, anti-Model, and anti-MOS (Figure 1).

### 2.4. Sample Collection

The mice were fed and watered normally until the end of the experiment. At the end of the experiment, the mice were anesthetized with pentobarbital and euthanized after the blood was taken from the eyeball. Blood samples were left to stratify, then the serum was obtained by centrifugation at 4 °C and 1000× *g* for 15 min and stored at −80 °C. The liver was taken and weighed, wrapped in tin foil, and stored at −80 °C. Kidneys were weighed, partly wrapped in tin foil and stored at −80 °C, and partly placed in paraformaldehyde for H&E staining. Intestinal contents were taken in lyophilization tubes, and the intestines were sectioned and wrapped in tin foil and stored at −80 °C.

### 2.5. Detection Method

#### 2.5.1. Determination of Serum Biochemical Indicators

Serum UA and BUN levels, and XOD and ADA enzyme activities were measured according to the kit instructions using commercial kits (Jiancheng Biotech, Nanjing, China).

#### 2.5.2. Urine and Fecal Uric Acid Content Measurement

The urine of mice was diluted 10 times and urine UA content was determined using UA assay kit kits (Jiancheng Biotech, Nanjing, China). In brief, we weighed 0.05 g of fresh mouse feces accurately, added 0.45 mL of saline, homogenized the mixture, and centrifuged it at 1000× *g* for 10 min at 4 °C. The supernatant was taken to obtain a 10% fecal homogenate sample, and the fecal UA content was determined using UA assay kit kits (Jiancheng Biotech, Nanjing, China).

#### 2.5.3. Liver Enzyme Activity Assay

Liver tissue was homogenized in 150 mM ice-cold PBS (pH 7.2) containing 1 mM EDTANa2 to prepare a 10% liver homogenate, and this was centrifuged at 1000× *g* for 10 min at 4 °C. The supernatant was taken to test the XOD and ADA enzyme activity using commercial kits (Jiancheng Biotech, Nanjing, China). Protein levels were determined by the BCA kit (Epizyme, Shanghai, China) using Bicinchoninic Acid with bovine serum albumin as a standard. Values were normalized to liver total protein.

#### 2.5.4. Histological Examination of Kidney Tissue

Histology examinations of kidney tissues were conducted following a previous protocol [32]. In brief, kidney samples were immersed in 4% paraformaldehyde at 4 °C for 24 h, then samples received gradient dehydration and were embedded in paraffin for tissue sections. Paraffin blocks of the liver tissue were cut into 4-μm-thick sections, which were then stained with hematoxylin and eosin (H&E) staining according to the standard protocol. Pathological analysis of the kidney was performed by two pathologists who were blinded to the exposure status.

#### 2.5.5. Measurement of Renal Inflammation Indicators

The kidney tissue was homogenized in 150 mM ice-cold PBS (pH 7.2) containing 1 mM EDTANa2 to prepare a 10% kidney homogenate and centrifuged at 1000× *g* for 10 min at 4 °C. Supernatant was taken to detect the kidney levels of IL-1β, IL-12, and IL-18 according to the ELISA kit instructions (Calvin, Suzhou, China). 

### 2.6. RT-qPCR Analysis

The total RNA was isolated from the tissues according to the protocols described in the previous study [33]. The kidney and intestinal tissues were placed in grinding tubes, and the total RNA was extracted by adding the appropriate amount of TRIzol. Reverse transcription was performed according to the instructions of All-In-One RT MasterMix (Abmgood, Shanghai, China), and the obtained cDNA was stored at a low temperature. The gene sequences of XOD, ADA, GLUT9, and URAT1 were queried on NCBI, and the primer design and synthesis were performed afterward (Table 1). The mRNA levels of various genes in the liver, kidney, and intestinal tissues were determined by qPCR using BlasTaq™ 2X qPCR MasterMix kit (Abmgood, Shanghai, China) with GAPDH as the internal reference gene. The amplification program was set as follows: enzyme activation at 95 °C for 10 min; 40 cycles of denaturation at 95 °C for 15 s and annealing/extension at 60 °C for 60 s. Relative quantification was performed by the 2^−ΔΔCT^ method using group Normal expression as 1.

### 2.7. Western Blot Analysis

Western blot analyses of the kidney and intestine tissues were conducted following a previous protocol [34]. Kidney and intestine tissue proteins were extracted and protein concentrations were determined by the BCA protein assay kit (Epizyme, Shanghai, China). Thirty μg of total proteins were separated by 10% SDS-PAGE and transferred to PVDF membranes. The membranes were closed with 5% skim milk powder for 2 h, incubated with primary antibody at 4 °C overnight, and then incubated with secondary antibody. Bands were visualized using WesternBright ECL (ABclonal, Wuhan, China) with ChemiDoc XRS + Imaging System (Bio-Rad Laboratories, Inc., Hercules, CA, USA). The grayscale values of the bands were calculated and normalized to the grayscale values of β-actin.

### 2.8. Gut Microbiota Analysis

#### 2.8.1. Dilute Coating Culture of Fecal Bacteria

Fresh feces from mice in the Normal and Antibiotics groups were placed in 2 mL enzyme-inactivated centrifuge tubes and homogenized at low temperature with 1 mL sterile water. After gradient dilutions of the homogenate (gradients of 10^−1^, 10^−2^, 10^−3^, 10^−4^), the sample dilutions were spread on a Columbia blood agar cell medium and incubated overnight at 37 °C.

#### 2.8.2. SCFAs Content Measurement

The content of SCFAs in the colon contents was determined by gas chromatography as previously described and optimized [35]. The stool sample (about 0.15 g) was weighed, and 1000 μL and 50 μL of 50% sulfuric acid were added and then centrifuged at 5000× *g* for 10 min. In total, 500 μL of supernatant was added to 50 μL of internal standard diethylbutyric acid and 500 μL of anhydrous ethyl ether, respectively, and centrifuged at 10,000× *g* for 10 min. For the GC analysis, 1 μL of supernatant was used, and the selected column was Agilent CB-FFAP (30 m × 0.25 mm, 0.25 μm). The column temperature chamber was gradient-tempered with the following procedure: the initial temperature was 100 °C and maintained for 0.5 min; then the temperature was increased to 180 °C at 8 °C/min and maintained for 1 min; then increased to 200 °C at 20 °C/min and maintained for 5 min. The detector was a hydrogen flame ionization detector (FID) at 250 °C.

#### 2.8.3. 16S rRNA Sequencing of Gut Microbiota

The total genomic DNA in the samples was extracted using the CTAB method. The V3V4 region of the 16S rRNA gene was selected for PCR amplification, and PCR was performed using specific primers with the barcode, Phusion^®^ High-Fidelity PCR Master Mix with GC Buffer (New England Biolabs, Salisbury, The United Kingdom), and high-efficiency high fidelity enzymes, depending on the amplified region. A NEBNext^®^ Ultra™ IIDNA Library Prep Kit (New England Biolabs, Salisbury, The United Kingdom) was used for library construction, and the constructed libraries were subjected to Qubit and Q-PCR quantification; after the libraries were qualified, NovaSeq6000 was used for up-sequencing. The samples were split from the downstream data according to the barcode sequence and PCR amplification primer sequence, and the reads were spliced using FLASH (V1.2.11, http://ccb.jhu.edu/software/FLASH/ (accessed on 20 January 2022)) software to obtain Raw Tags after truncating the barcode and primer sequences. Finally, the Clean Tags were compared with the database using Usearch software to detect chimeras and remove them to obtain the final validated data, i.e., Effective Tags. The final ASVs (Amplicon Sequence Variants) and feature tables were obtained by noise reduction using the DADA2 module of QIIME2 software and filtering out the sequences with an abundance of less than 5. Subsequently, the obtained ASVs were compared with the database using the classify-sklearn module in QIIME2 software to obtain the specific information of each ASV. Unifrac distances were calculated using QIIME2 software, and R software was used to plot the NMDS downscaling. The significance of the differences in community structure between the groups was analyzed using the pairwise PERMANOVA. Significantly different species analysis between the groups was completed using DEseq2. Among them, the DEseq2 analysis was performed by R software.

### 2.9. Statistical Analysis

Data were represented as the mean ± standard error of the mean (SEM). Normality was tested by the Shapiro–Wilk test. For parametric variables, differences between the two groups were assessed using the unpaired two-tailed Student’s *t*-test. Data sets that involved more than two groups were assessed by one-way ANOVA followed by Newman–Keuls post hoc tests. For nonparametric variables, the statistical significance of differences in bacterial relative proportion among the study groups was tested by the nonparametric Kruskal–Wallis test followed by the Mann–Whitney U test when *p* < 0.05. SPSS22.0 software (Chicago, IL, USA) and GraphPad Prism software (Version 9.2) were used to perform the statistical analysis.

## 3. Results

### 3.1. MOS Treatment Alleviated Hyperuricemia in Mice

**Figure 2:**
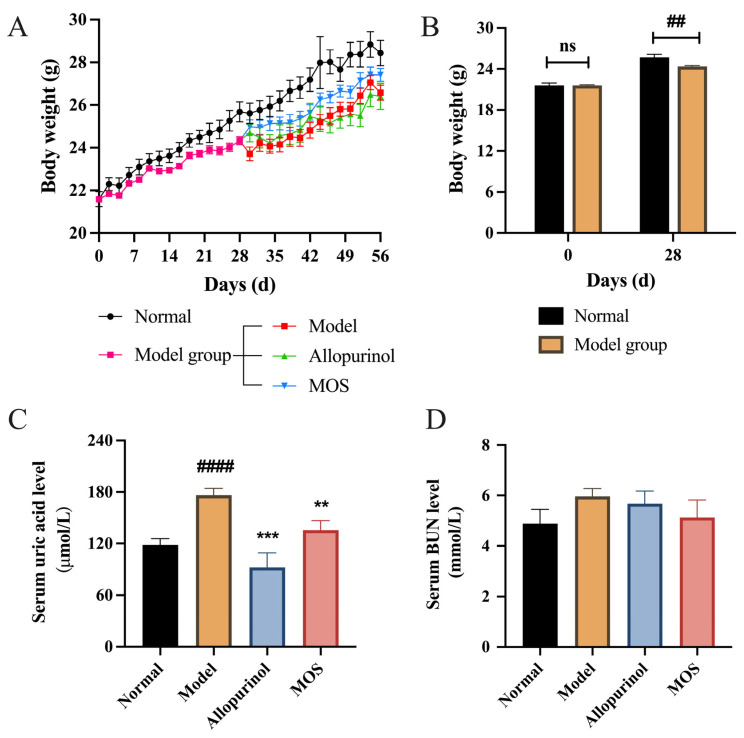
Ameliorative effect of MOS on HUA in mice. Body weight (**A**); Weight gain in mice during modeling (**B**); Serum UA (**C**); Serum BUN (**D**). ## *p* < 0.01, and #### *p* < 0.0001 versus normal. ** *p* < 0.01, and *** *p* < 0.001 versus model. The ‘ns’ indicates no statistical difference.

First, potassium oxyzincate was intraperitoneally injected into mice for 4 weeks to construct the hyperuric acid mouse model. The mice’s body weight rose continuously during the duration of the study (Figure 2A,B). Hyperuricemia is a disorder of purine metabolism that is characterized by an above-normal serum UA level owing to increased UA production or reduced serum UA excretion. At the end of the fourth week, the serum uric acid level of mice in the modeling group was substantially greater than that of mice in the Normal group, showing that the hyperuricemia animal model was established effectively. The effectively modeled mice were then randomly allocated into three groups: model, positive drug, and MOS treatment. The levels of serum uric acid and body weight of mice in the model group, the positive drug group, and the MOS treatment groups were comparable (Supplementary Figure S1).

Subsequently, treatment was carried out on the mice. After 4 weeks of treatment, MOS significantly decreased serum uric acid levels compared to the Model group (Figure 2C, *p* < 0.01). Frequently, hyperuricemia is accompanied with alterations in renal function, and the quantity of blood urea nitrogen (BUN) is a crucial sign of these alterations. In hyperuricemic mice, the blood BUN level rose from 4.89 mmol to 5.96 mmol, however, MOS treatment decreased the serum BUN level from 5.96 mmol to 5.13 mmol, which is near its level in normal animals, but not significant (Figure 2D). These results indicated that MOS could relieve the symptoms of high UA and be expected as an effective agent for the treatment of hyperuricemia.

### 3.2. MOS Does Not Affect the Synthesis of Uric Acid

**Figure 3:**
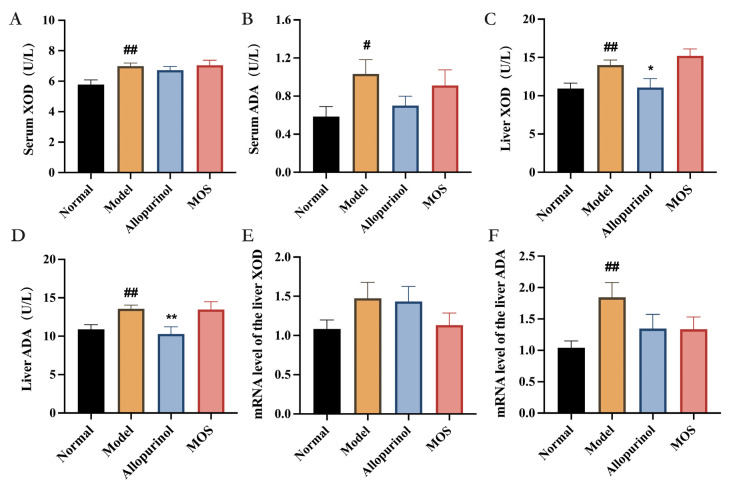
The effect of MOS on uric acid synthesis in HUA mice. Serum XOD activity (**A**); Serum ADA activity (**B**); Liver XOD activity (**C**); Liver ADA activity (**D**); mRNA levels of liver XOD (**E**); mRNA levels of liver ADA (**F**). # *p* < 0.05, and ## *p* < 0.01 versus normal. * *p* < 0.05, and ** *p* < 0.01 versus model.

The overproduction of uric acid was one of the main causes of hyperuricemia. Purines from food intake are further processed in the liver into uric acid. The two enzymes, Xanthine oxidase (XOD) and Adenosine deaminase (ADA), are essential for UA production, which converts purines into uric acid. The levels of XOD and ADA in serum and hepatic tissues were found to be increased after induction of hyperuricemia, whereas the activities of hepatic XOD and ADA were significantly decreased by allopurinol but unaffected by MOS. Moreover, the results of qRT-PCR also confirmed that MOS did not significantly affect XOD and ADA at mRNA levels in the liver. These findings imply that MOS may not reduce serum uric acid levels by influencing hepatic uric acid synthesis (Figure 3).

### 3.3. MOS Alleviates Kidney Injuries by Reducing Kidney Inflammation

**Figure 4:**
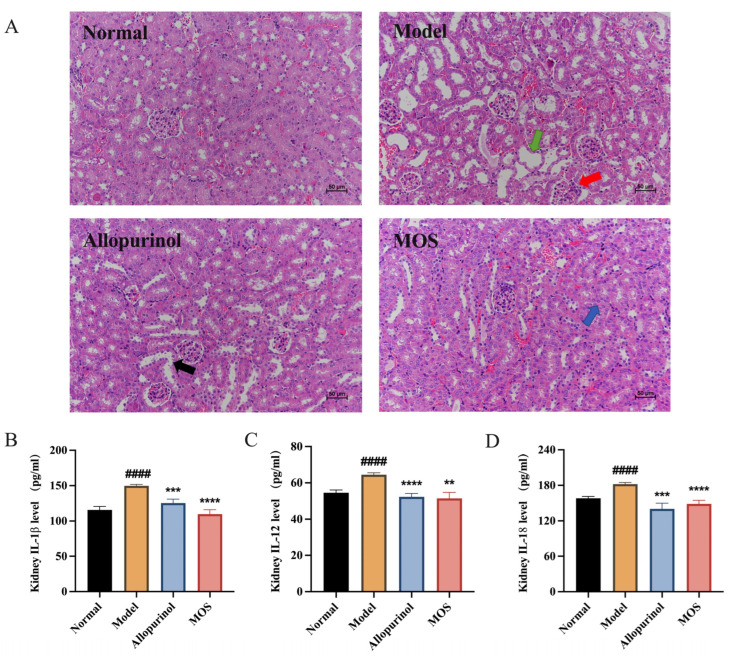
The ameliorative benefits of MOS on renal injuries. Renal H&E-stained sections with a magnification of 200× (**A**); IL1β (**B**); IL-12 (**C**); IL-18 (**D**). Green arrow: renal tubular dilatation, red arrow: inflammatory cell infiltration, black arrow: epithelial cell detachment, blue arrow: attenuated inflammatory cell infiltration. #### *p* < 0.0001 versus normal. ** *p* < 0.01, *** *p* < 0.001, and **** *p* < 0.0001 versus model.

The development of hyperuricemia is often accompanied with altered renal pathology. H&E staining of renal sections from the Model group showed visible vacuolation, glomerular atrophy, and tubular dilatation (green arrow) accompanied by inflammatory cell infiltration (red arrow), whereas the allopurinol group showed glomerular atrophy and tubular dilatation with epithelial cell detachment (black arrow), suggesting that allopurinol may have toxic effects on the kidney. MOS treatment ameliorated renal pathology and attenuated inflammatory cell infiltration (blue arrows) among hyperuricemic mice. Proinflammatory cytokines play a critical role in the progression of hyperuricemia-induced kidney injury. Compared with the Normal group, the levels of the pro-inflammatory factors IL1β, IL-12, and IL-18 were significantly increased in the Model mice, while they were dramatically lowered by MOS treatment. These data suggest that MOS treatment suppresses the inflammatory response in hyperuricemia-induced kidney inflammation (Figure 4).

### 3.4. MOS Promotes Uric Acid Excretion

**Figure 5:**
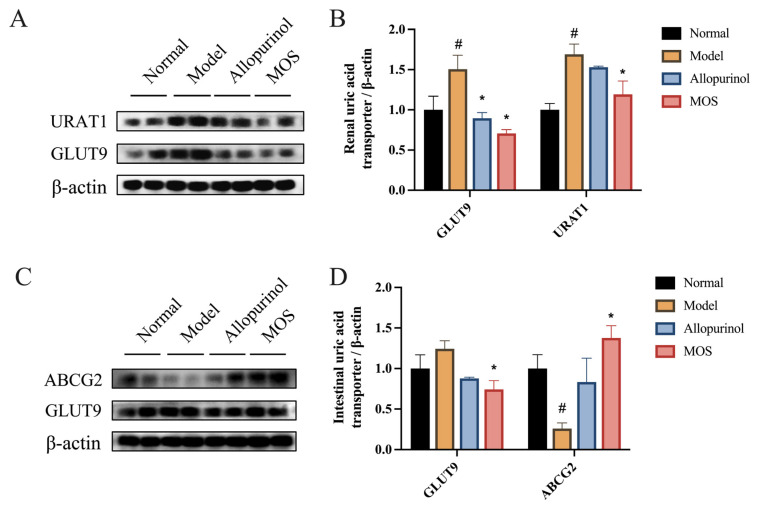
Improvement of uric acid excretion by MOS. Renal URAT1 and GLUT9 protein expression (**A**); Quantitative analysis of renal URAT1 and GLUT9 protein (**B**); Intestinal ABCG2, GLUT9 protein expression (**C**); Quantitative analysis of intestinal ABCG2, GLUT9 protein (**D**). # *p* < 0.05 versus normal. * *p* < 0.05 versus model.

Inadequate excretion of uric acid is another cause of hyperuricemia in addition to excessive purine synthesis. In this study, we did not find a significant effect of MOS on the uric acid synthesis pathway in the liver (Figure 3). Therefore, we hypothesize that MOS may improve hyperuricemia by acting on the uric acid excretion process. To confirm the effect of MOS on the process of uric acid excretion, we analyzed the mRNA and protein expression levels of regulatory factors of uric acid excretion in the kidney and intestine. GLUT9 and URAT1 are the major uric acid transporters in the kidney. MOS treatment downregulated the protein levels of renal URAT1 and GLUT9, and the changes in mRNA levels confirmed the regulatory effect of MOS. GLUT9 and ABCG2 are the main uric acid transporters in the intestine, and the results showed that MOS downregulated intestinal GLUT9 and upregulated intestinal ABCG2 (Figure 5, Supplementary Figure S2). The results revealed that MOS may promote renal and intestinal excretion of uric acid.

### 3.5. MOS Alters the Structure of Gut Microbiota in HUA Mice

**Figure 6:**
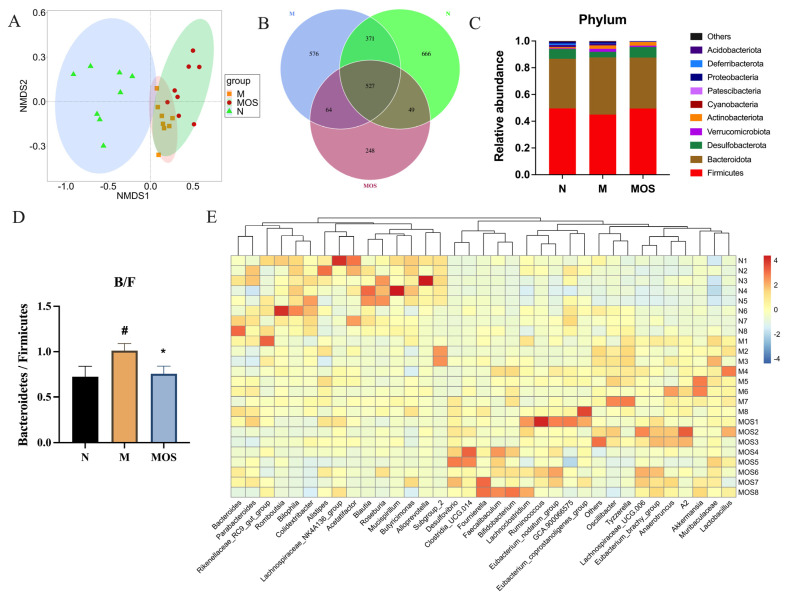
Alterations in intestinal flora structure and composition under MOS treatment. NMDS analysis (**A**); Venn diagram of ASVs (**B**); Changes in gut flora structure at the gate level (**C**); The Bacteroidetes/Firmicutes ratio (**D**); Relative abundance at the genus level shown as a heat map (**E**). N: Normal group, M: Model group. # *p* < 0.05 versus normal. * *p* < 0.05 versus model. N: Normal group, M: Model group.

Changes in the gut microbiota are directly related to the development of hyperuricemia [36]. The composition of the caecum gut microbiota was determined by standard 16S rRNA sequencing. The NMDS analysis demonstrates that the makeup of the gut microbiota of mice varied significantly between the three groups (Figure 6A). Furthermore, the PERMANOVA test further demonstrated that gut microbiota structures were considerably changed by MOS treatment (Supplementary Table S1). The Venn diagram showed 527 ASVs common to all three groups and 297 ASVs in the MOS group were not found in the Model group (Figure 6B). At the phylum levels, the microbiota was found to be mainly composed of *Firmicutes*, *Bacteroidetes*, *Actinobacteria*, and *Proteobacteria* (Figure 6C). The ratio of Bacteroidetes/Firmicutes, an imbalance of which may lead to metabolic syndrome [37], was almost restored to the level of the Normal group of mice by MOS treatment (Figure 6D). Furthermore, the gut microbiota has different species composition at the genus level (Figure 6E). Taken together, these findings indicate that MOS supplementation modifies the composition and structure of the microbiota in hyperuricemic mice.

### 3.6. MOS Increased Beneficial Bacteria and Reduced the Abundance of Harmful Bacteria

**Figure 7:**
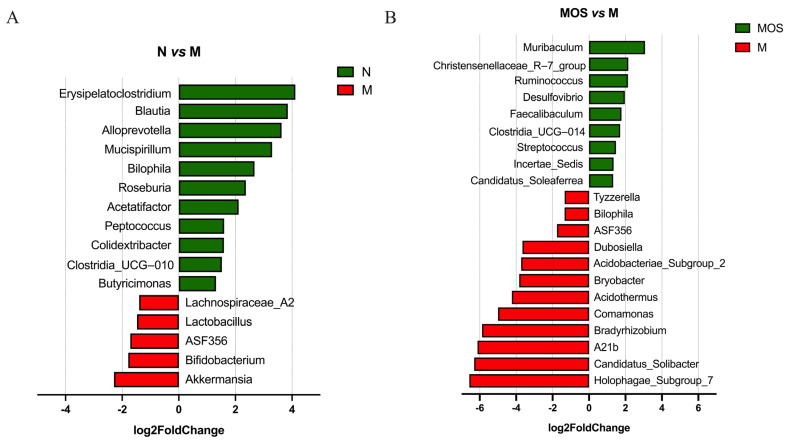
DEseq2 analysis of differential species. N vs. M (**A**); MOS vs. M (**B**). All data shown are consistent with *p* < 0.05 and a Fold change > 2. N: Normal group, M: Model group.

To determine the effect of MOS supplementation on intestinal microorganisms in hyperuricemic mice, the DEseq2 analysis was employed to identify major differential biomarkers among the microbiota. At the genus level, the abundance of several beneficial bacteria was markedly downregulated in the Model group, including *Butyricimonas*, *Clostridia_UCG−010*, *Roseburia*, *Mucispirillum*, *Alloprevotella*, *Blautia*, which are associated with short-chain fatty acids production, and anti-inflammatory-related genera *Colidextribacter* [38]. Compared to the Model group, the levels of *Tyzzerella*, *Bilophila*, *ASF356*, *Bryobacter*, *Comamonas*, and *Candidatus_Solibacter* were downregulated in the MOS group. Meanwhile, the abundance of beneficial bacteria was obviously upregulated, including *Muribaculum*, *Ruminococcus*, *Faecalibaculum*, *Christensenellaceae_R−7_group*, *Candidatus_Soleaferrea*, and *Clostridia_UCG−014* (Figure 7, Supplementary Figure S3). 

### 3.7. Changes in Metabolic Parameters of HUA Mice Correlate with Intestinal Flora

**Figure 8:**
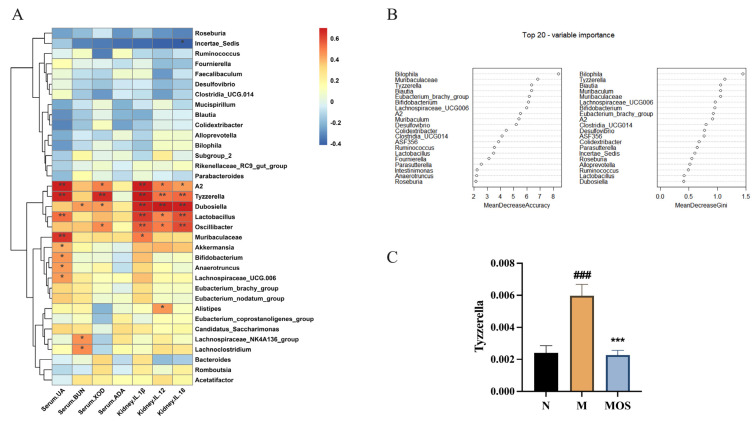
Changes in metabolic parameters of HUA mice correlate with gut microbiota. Spearman’s correlation analysis between intestinal flora and metabolic parameters (**A**); The Random Forest (**B**); Abundance of *Tyzzerella* (**C**). In plot A, * *p* < 0.05, ** *p* < 0.01. In plot C, ### *p* < 0.001 versus normal, *** *p* < 0.001 versus model. N: Normal group, M: Model group.

To examine the association between changes in gut bacteria and hyperuricemia remission, Spearman’s correlation analysis was employed to correlate bacteria and hyperuricemia-related metabolic variables at the genus level. The results showed that *Tyzzerella*, *Lactobacillus*, *Dubosiella*, *A2*, and *Muribaculaceae* presented strong positive correlations with uric acid and proinflammation factors, such as IL-12, IL-1β and IL-18. *Incertae_Sedis* presented strong negative correlations with renal IL-1β. These findings imply that variations in the levels of bacteria *Tyzzerella*, *Lactobacillus*, *Dubosiella*, *A2*, and *Muribaculaceae* may have outstanding value for variations in the variables influencing hyperuricemia-related effects. The Random Forest (RF) was used to estimate the correlation between hyperuricemia-related effects and important microbial taxa. As Figure 8B shows, the top 20 genera provided excellent accuracy in classifying Model and Normal mice. The genus *Tyzzerella*, ranked as the third most important feature, based on mean decrease accuracy, was found to be about three-fold more abundant in the Model mice than in Normal mice. In addition, the genus *Tyzzerella* showed a strong positive correlation with hyperuricemia-related effects. Compared with the Model, the MOS supplement significantly lowered the levels of *Tyzzerella*. These findings imply that MOS supplementation may have improved the hyperuricemia phenotype in mice via influencing *Tyzzerella* levels (Figure 8). 

### 3.8. MOS Treatment Induces Metabolic Functional Changes in the Gut Microbiota of HUA Mice

**Figure 9:**
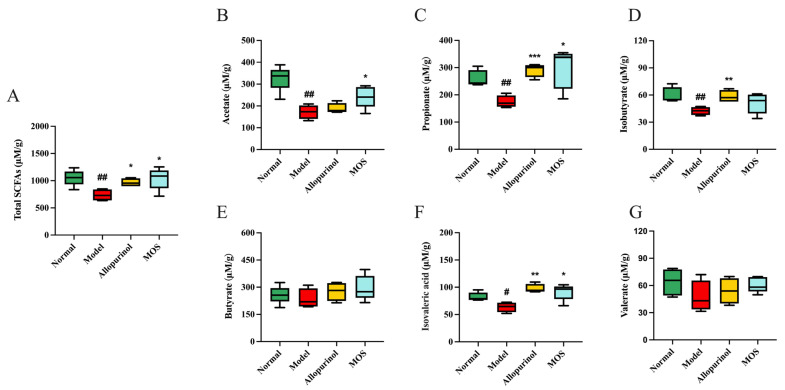
Effect of MOS treatment on SCFAs. Total SCFAs content (**A**); Acetic acid (**B**); Propionic acid (**C**); Isobutyric acid (**D**); Butyric acid (**E**); Isovaleric acid (**F**); Valeric acid (**G**). # *p* < 0.05, and ## *p* < 0.01 versus normal. * *p* < 0.05, ** *p* < 0.01, and *** *p* < 0.001 versus model.

SCFAs are metabolites of intestinal bacteria, and their changes represent the changes in the metabolic function of the gut microbiota. SCFAs can improve the morphology and function of intestinal epithelial cells, and thus, have an effect on intestinal uric acid transporters [39]. In this study, the levels of SCFAs in the colon contents were detected by gas chromatography (GC). In the Model group, the level of SCFAs was lower than that of the Normal group, among which acetic acid, propionic acid, isobutyric acid, and isovaleric acid varied significantly (*p* < 0.05). After MOS treatment, the levels of SCFAs increased, with significant changes in acetic acid, propionic acid, and isovaleric acid. The results indicate that MOS modulated the gut microbiota, leading to an increased production of SCFAs and improved morphology and function of intestinal epithelial cells. Subsequently, the quantity, distribution, and function of intestinal uric acid transporters in the hyperuricemic mice were altered (Figure 9).

### 3.9. Antibiotics Can Eliminate the Effect of MOS

**Figure 10:**
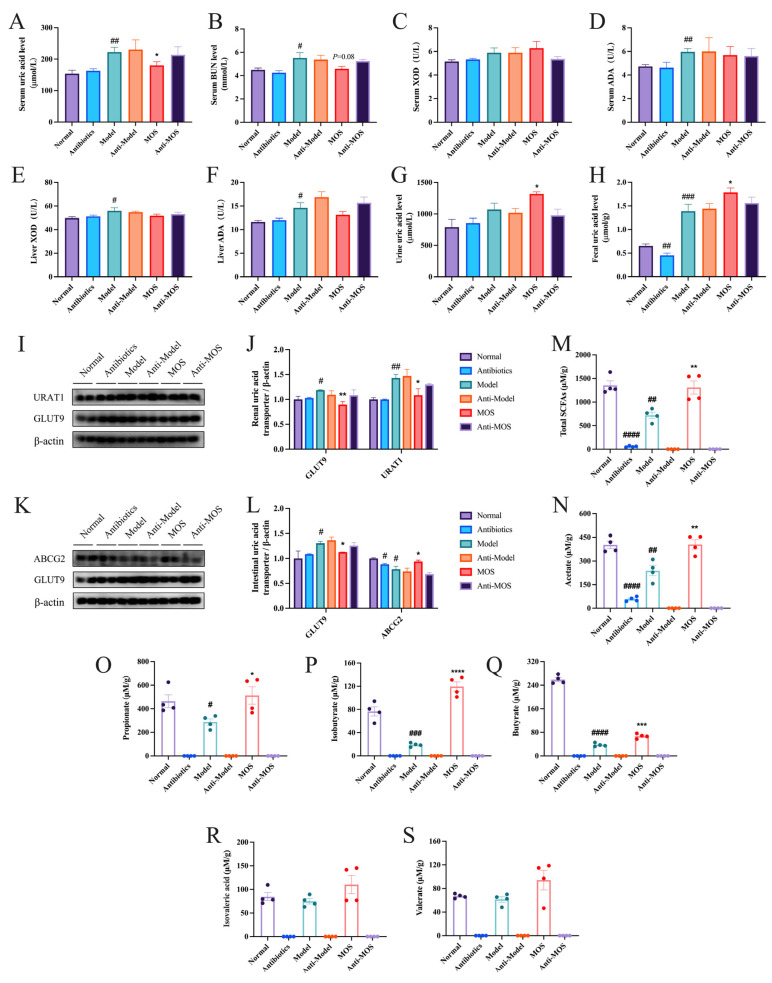
Effect of MOS on antibiotic-treated mice. Serum uric acid (**A**); Serum BUN (**B**); Serum XOD activity (**C**); Serum XOD activity (**D**); Hepatic XOD activity (**E**); Hepatic ADA activity (**F**); Urinary uric acid (**G**); Fecal uric acid (**H**); Renal URAT1 and GLUT9 protein expression (**I**); Quantitative analysis of renal URAT1 and GLUT9 protein (**J**); Intestinal ABCG2, GLUT9 protein expression (**K**); Quantitative analysis of intestinal ABCG2, GLUT9 protein (**L**); Total SCFAs content (**M**); Acetic acid (**N**); Propionic acid (**O**); Isobutyric acid (**P**); Butyric acid (**Q**); Isovaleric acid (**R**); Valeric acid (**S**). # *p* < 0.05, ## *p* < 0.01, ### *p* < 0.001, and #### *p* < 0.0001 versus normal. * *p* < 0.05, ** *p* < 0.01, *** *p* < 0.001, and **** *p* < 0.0001 versus model.

To verify that MOS has the ability to regulate gut microbiota in HUA mice, we used antibiotic-treated mice for further study. After antibiotic administration, the mice were largely cleared of intestinal microorganisms (Supplementary Figure S4). With the eradication of intestinal bacteria, the impact of MOS in reducing hyperuricemia in mice disappeared, while the antibiotic had no discernible effect on uric acid levels in the animals (Figure 10A, Supplementary Figure S5). These findings imply that the impact of MOS on reducing hyperuricemia in mice is reliant on the gut bacteria. 

In uric acid synthesis, serum and liver XOD and ADA enzyme activity levels were elevated in HUA mice, but without any significant effect of MOS (Figure 10C–F). In uric acid excretion, urinary UA levels were elevated by 35.33% and fecal UA by 113.8% in the Model group, and MOS treatment significantly increased urinary and fecal UA levels. In addition, renal URAT1, GLUT9 and intestinal GLUT9 protein levels were significantly elevated and intestinal ABCG2 protein levels were significantly decreased in HUA mice, and MOS treatment reversed the protein levels of these uric acid transporters. This result is consistent with the results of the first experiments. There was no significant difference between the Anti-MOS and Model groups (Figure 10G–L). These findings provide additional evidence that MOS alleviates hyperuricemia in mice through increasing uric acid excretion and not by changing uric acid production.

The content of SCFAs in the colon contents was further measured. The results revealed that the content of SCFAs in the Model group was obviously lower than that in the Normal group, and the content of SCFAs recovered after MOS treatment, as well as the SCFAs were basically undetectable in mice with antibiotics (Figure 10M–S). In review, MOS significantly reduced serum UA levels and promoted uric acid excretion, and the use of antibiotics largely eliminated the effect of MOS, indicating that intestinal flora has an important role in the uric acid excretion promotion of MOS.

## 4. Discussion

Due to modern lifestyle changes, the prevalence of hyperuricemia is rising every year and has been the second most common metabolic disease followed by diabetes [40]. In this study, we investigated the anti-hyperuricemia effect and mechanism of mannuronate oligosaccharides (MOS). Our research showed that MOS ameliorates renal inflammation and promotes renal and intestinal uric acid excretion in hyperuricemic mice. In addition, the changes in gut microbiota during the alleviation of hyperuricemia by MOS treatment were investigated, and MOS was confirmed to regulate the composition and abundance of gut microbiota. It was further validated by the antibiotic-treated mouse experiment that the anti-hyperuricemia and anti-inflammatory effects of MOS were mediated partly via intestinal bacteria. 

The progression of hyperuricemia is usually accompanied by kidney injury. The kidneys are the primary organ for uric acid excretion, and it is vital for the body to maintain normal renal function. Excessive uric acid production, or inadequate uric acid excretion, can lead to uric acid deposits in the kidneys, leading to decreased kidney function and renal inflammation [41]. Serum BUN is an indicator of renal function, and the levels of pro-inflammatory cytokines IL-1β, IL-12, and IL-18 in the kidney also represent the degree of renal impairment [42,43,44]. It was demonstrated that in hyperuricemic mice, serum BUN was increased and renal pro-inflammatory factors IL-1β, IL-12 and IL-18 were significantly elevated [45,46]. Meanwhile, Chen et al. [47] observed pathological changes such as glomerular atrophy, tubular dilatation, and renal inflammatory cell infiltration in hyperuricemic mice. During this study, serum BUN, and renal IL-1β, IL-12 and IL-18 levels in mice fed a high purine diet and injected with potassium oxonate intraperitoneally, were consistent with the index changes observed by Wu et al. [45] in HUA mice. In addition, the hyperuricemic mice in this study observed similar renal pathological changes as in the study of Zhang et al. [48]. Obviously, the trend of MOS on these indicators is consistent with the effects presented by inulin and chicory [14,49], which have been reported to have a positive effect on the treatment of hyperuricemia, and MOS improved the renal lesions better than allopurinol in this study. The kidneys of hyperuricemic mice were injured by uric acid-induced inflammation, which was healed within four weeks by MOS administration, indicating that MOS may have a protective impact on the kidneys. MOS’s suppressed inflammatory response and reduction of renal pathological changes were the primary causes of hyperuricemia improvement.

Maintaining the balance of uric acid biosynthesis and excretion is essential for uric acid metabolism. In clinical practice, drugs used to treat hyperuricemia work by inhibiting uric acid synthesis or promoting uric acid excretion, such as allopurinol and febuxostat, which are XOD inhibitors that depress uric acid synthesis [50], in contrast to benzbromarone and probenecid, which promote uric acid excretion by suppressing URAT1 and GLUT9 [51]. Uric acid is synthesized in the liver, and XOD and ADA enzymes are key enzymes in the uric acid production pathway. XOD enzyme catalyzes the oxidation of hypoxanthine to xanthine, then to UA; ADA enzyme catalyzes the conversion of adenine nucleoside to hypoxanthine nucleoside, and its metabolic end product is also UA [52]. In our study, XOD and ADA enzyme activities were significantly elevated in mice suffering from hyperuricemia, which is consistent with the findings of Le et al. [53,54]. In contrast, astaxanthin decreased XOD and ADA enzyme activities and inhibited uric acid synthesis in HUA mice in the study by Le et al. [54]. Regrettably, we did not observe changes in XOD and ADA enzyme activities after MOS treatment, indicating that MOS may be anti-hyperuricemia not by affecting uric acid synthesis, but by influencing uric acid excretion. The excretion of uric acid is another important factor in the pathogenesis of hyperuricemia. The excretion of uric acid relies on uric acid transporters, of which URAT1 and GLUT9 are responsible for uric acid reabsorption, while ABCG2 plays a key role in urate re-secretion [55,56]. These uric acid transporters are also potential targets for the treatment of hyperuricemia. Chau et al. found that the mRNA and protein expression levels of URAT1 and GLUT9 were upregulated, and those of ABCG2 were downregulated in hyperuricemic mice [57,58]. In our study, we also observed similar changes in HUA mice. The reported natural active substances with excellent anti-hyperuricemia effects have modulating effects on urate transporters. For example, fucoidan and fucoxanthin promote the renal excretion of uric acid by regulating the mRNA and protein expression of renal GLUT9, ABCG2, and URAT1 [58]; stevia residue extract promotes the intestinal excretion of uric acid by regulating the protein expression of intestinal GLUT9 and ABCG2 [57]. In this study, the modulatory effects of MOS on renal URAT1, GLUT9, intestinal GLUT9, and ABCG2 were consistent with the effects of the anti-hyperuricemic substance fucoidan and stevia residue extract in the literature [57,58]. This result reveals the mechanism of the UA-reducing effect of MOS: it may be regulating renal and intestinal urate transporters, thereby promoting UA excretion in the kidney and intestine.

Recent researches have found a close association between intestinal flora and hyperuricemia. It was found that HUA mice and normal mice have significantly different intestinal flora composition and structure [59]. This situation was also observed during our study. Furthermore, Pan et al. [60] reported that intestinal flora influenced the process of uric acid excretion. Li et al. [61] investigated how the excretion of uric acid could be altered by regulating intestinal flora, hence alleviating hyperuricemia. Dysregulation of the nitrogen cycle in the intestinal flora causes worsening renal damage, which further impairs HUA [62]. This means that the intestinal flora can influence the renal excretion of uric acid. The intestine is another route of uric acid excretion, and an increase of UA in the blood circulation leads to a large amount of UA secreted into the intestinal lumen, causing gut flora disorders. In this case, the intestinal flora aggravates intestinal inflammation via intermediate metabolites and then affects the distribution and number of uric acid transporters, further escalating HUA [63]. This means that the intestinal flora affects the intestinal excretion of uric acid. Our findings implied that MOS improved hyperuricemia by altering uric acid excretion. Accordingly, we focused on the role of intestinal flora in regulating the process of hyperuricemia.

MOS-treated mice and HUA mice had a significantly different intestinal flora composition and structure. Consistent with the results of previous studies, the relative abundance of *Firmicutes* was observed to decrease and the relative abundance of *Bacteroidetes* increased in HUA mice [49]. The elevated *Bacteroidetes*/*Firmicutes* ratio is a feature of microbiota disruption induced by hyperuricemia, and the anti-hyperuricemia probiotics *Lactobacillus plantarum* Q7 and X11 reduce the *Bacteroidetes*/*Firmicutes* ratio and modulate the intestinal flora structure in HUA mice [64,65]. A similar change was observed in this study, whereby MOS supplementation reversed the *Bacteroidetes*/*Firmicutes* ratio. At the genus level, the abundance of the SCFAs production-associated genera *Butyricimonas*, *Clostridia_UCG−010*, *Roseburia*, *Mucispirillum*, *Alloprevotella*, and *Blautia* decreased with the increasing UA levels in the Model group, and this result is consistent with our finding that the SCFAs content was reduced. In addition, *Colidextribacter* and *Bilophila* abundance decreased in the Model group, while *Akkermansia* was upregulated. Among these bacteria, *Colidextribacter* was able to produce inosine, which inhibits the production of pro-inflammatory cytokines and chemokines as well as promotes the production of anti-inflammatory cytokines [66]. Wu et al. [67] found a significant decrease in the abundance of *Blautia*, *Colidextribacter* in hyperuricemic mice, which is consistent with the changes we observed. MOS modified the altered microbiota of hyperuricemic mice. The abundance of pathogenic bacteria *Bilophila*, *Tyzzerella*, *Bryobacter*, *Comamonas*, and *Candidatus_Solibacter* was significantly downregulated in the MOS group [68,69,70]. *Tyzzerella* was strongly positively associated with the risk of high cardiovascular risk and obesity-related diseases [71,72]. In this study, *Tyzzerella* was found to present a strong positive correlation with pro-inflammatory factors, which was in agreement with the findings of Grant et al. [73]. Meanwhile, *Tyzzerella* was considered a key marker in Random Forest analysis to distinguish between different physiological states. *Tyzzerella* was significantly increased in HUA mice, while MOS could effectively reverse this trend. Such information suggests that *Tyzzerella* may be an important way for MOS to regulate intestinal flora, and thus, enhance uric acid excretion. Besides the decrease in the abundance of harmful bacteria, MOS also promoted the proliferation of beneficial bacteria, such as *Muribaculum*, *Ruminococcus,* and *Faecalibaculum*, which are associated with short-chain fatty acid production, and the anti-inflammatory-related bacteria *Christensenellaceae_R−7_group* and *Candidatus_Soleaferrea*, etc. [74,75]. In the study of Liang et al. [76], the abundance of *Ruminococcus* in the intestinal flora of HUA patients was significantly decreased and was considered a microbiological marker of HUA, while in the present study, this bacterium could be upregulated by MOS.

To further confirm the important role played by intestinal flora in the amelioration of hyperuricemia by MOS treatment, we additionally explored with antibiotic-treated mice. The uric acid-lowering effect of MOS in HUA mice was diminished following the elimination of gut microbes. Moreover, MOS treatment did not significantly affect the uric acid synthesis pathway (hepatic ADA and XOD enzyme activity) in the HUA mice with culled flora. The urine and fecal uric acid levels, which are important indicators of the uric acid excretion process, were significantly higher in the feces and urine of MOS-intervened mice compared to the Anti-MOS group after flora elimination. Moreover, the modulation of renal and intestinal uric acid transporters by MOS was consistent with the results of the first animal experiments, and the effect of MOS on uric acid transporters was eliminated by antibiotics. This provides a further indication that MOS facilitates the process of uric acid excretion through gut microbiota, and thus, ameliorates hyperuricemia in mice.

In this work, the effect of MOS on reducing hyperuricemia in mice and the probable mechanisms behind this response were investigated. The results will be helpful for the development of natural products and will serve as a resource for targeting the management of gut microbiota to alleviate metabolic syndrome. However, there are still some limitations to our study: we did not pursue the molecular processes by which gut microbiota influences hyperuricemia, and furthermore, although MOS was verified to increase uric acid levels in urine and feces in the second experiment, uric acid excretion was not measured in the first experiment. This demands further investigation into the underlying mechanics.

## 5. Conclusions

In conclusion, MOS can alleviate HUA, relieve renal inflammation and modulate gut microbiota in HUA mice. The hypouricemic effect of MOS is attributed to its modulation of urate transporters in the kidney and gut, thus promoting UA excretion. Alleviation of renal inflammation by MOS is mediated by the inhibition of pro-inflammatory cytokines. The MOS intervention and antibiotic-treated mice experiments directly demonstrated that MOS can modulate the gut microbiota of HUA mice, and its anti-hyperuricemia effect is dependent on and regulated by the gut flora. In this study, we investigated the effect and mechanism of MOS to alleviate HUA, and the results suggest that MOS may be a potential candidate for the treatment of HUA.

## Figures and Tables

**Table 1:** Gene primer sequences.

Gene	Primers	Sequence
XOD	FORWARD	TTCAAACCTTTAGATCCCACCC
	REVERSE	GGGCAGATGATCAGAGGAAATA
ADA	FORWARD	TCTATGTGGAAGTGCGCTATAG
	REVERSE	TTCACAAGATCCACAACGTCAT
GLUT9	FORWARD	ATGTGGACTCAATGCGATCTGGTTC
	REVERSE	TGTTTCAATTCCTCCCGTGCTCAG
URAT1	FORWARD	GACCTTGGACCCGATGTTCTTCTG
	REVERSE	CGTGGCGTTGGACTCTGTAAGC

## Data Availability

Data is contained within the article and can be available upon request.

## Supplementary Materials

The following supporting information can be downloaded at: https://www.mdpi.com/article/10.3390/nu15020417/s1. Figure S1: Serum uric acid levels after four weeks of induced hyperuricemia in the first experiments; Figure S2: Effect of MOS on mRNA levels of uric acid transporters; Figure S3: Microbial taxa discrepancies among Normal, Model, and MOS groups (LDA scores of >2.5 and adjusted *p* values of <0.05); Figure S4: Number of colonies in feces in the second experiments; Figure S5: Serum uric acid levels after four weeks of induced hyperuricemia in the second experiments; Table S1: The variability of the intestinal flora structure between the groups.

## References

[1] Song P., Wang H., Xia W., Chang X., Wang M., An L. (2018). Prevalence and correlates of hyperuricemia in the middle-aged and older adults in China. Sci. Rep..

[2] Yokose C., McCormick N., Choi H.K. (2021). The role of diet in hyperuricemia and gout. Curr. Opin. Rheumatol..

[3] Butler F., Alghubayshi A., Roman Y. (2021). The Epidemiology and Genetics of Hyperuricemia and Gout across Major Racial Groups: A Literature Review and Population Genetics Secondary Database Analysis. J. Pers. Med..

[4] Huang J., Ma Z.F., Zhang Y., Wan Z., Li Y., Zhou H., Chu A., Lee Y.Y. (2020). Geographical distribution of hyperuricemia in mainland China: A comprehensive systematic review and meta-analysis. Glob. Health Res. Policy.

[5] Borghi C., Agabiti-Rosei E., Johnson R.J., Kielstein J.T., Lurbe E., Mancia G., Redon J., Stack A.G., Tsioufis K.P. (2020). Hyperuricaemia and gout in cardiovascular, metabolic and kidney disease. Eur. J. Intern. Med..

[6] Bobulescu I.A., Moe O.W. (2012). Renal transport of uric acid: Evolving concepts and uncertainties. Adv. Chronic Kidney Dis..

[7] Ichida K., Matsuo H., Takada T., Nakayama A., Murakami K., Shimizu T., Yamanashi Y., Kasuga H., Nakashima H., Nakamura T. (2012). Decreased extra-renal urate excretion is a common cause of hyperuricemia. Nat. Commun..

[8] Zhu C., Sun B., Zhang B., Zhou Z. (2021). An update of genetics, co-morbidities and management of hyperuricaemia. Clin. Exp. Pharmacol. Physiol..

[9] Song D., Zhao X., Wang F., Wang G. (2021). A brief review of urate transporter 1 (URAT1) inhibitors for the treatment of hyperuricemia and gout: Current therapeutic options and potential applications. Eur. J. Pharmacol..

[10] Wang Z., Cui T., Ci X., Zhao F., Sun Y., Li Y., Liu R., Wu W., Yi X., Liu C. (2019). The effect of polymorphism of uric acid transporters on uric acid transport. J. Nephrol..

[11] Kim S., Kim H.J., Ahn H.S., Oh S.W., Han K.H., Um T.H., Cho C.R., Han S.Y. (2017). Renoprotective effects of febuxostat compared with allopurinol in patients with hyperuricemia: A systematic review and meta-analysis. Kidney Res. Clin. Pract..

[12] Jansen T.L., Tanja G., Matthijs J. (2022). A historical journey of searching for uricosuric drugs. Clin. Rheumatol..

[13] Tatrai P., Erdo F., Dornyei G., Krajcsi P. (2021). Modulation of Urate Transport by Drugs. Pharmaceutics.

[14] Bian M., Wang J., Wang Y., Nie A., Zhu C., Sun Z., Zhou Z., Zhang B. (2020). Chicory ameliorates hyperuricemia via modulating gut microbiota and alleviating LPS/TLR4 axis in quail. Biomed. Pharmacother..

[15] Zhang Y., Tan X., Lin Z., Li F., Yang C., Zheng H., Li L., Liu H., Shang J. (2021). Fucoidan from Laminaria japonica Inhibits Expression of GLUT9 and URAT1 via PI3K/Akt, JNK and NF-kappaB Pathways in Uric Acid-Exposed HK-2 Cells. Mar. Drugs.

[16] Li X., Gao X., Zhang H., Liu Y., Sarker M.M.R., Wu Y., Chen X., Zhao C. (2021). The anti-hyperuricemic effects of green alga Enteromorpha prolifera polysaccharide via regulation of the uric acid transporters in vivo. Food Chem. Toxicol..

[17] Shen P., Gu Y., Zhang C., Sun C., Qin L., Yu C., Qi H. (2021). Metabolomic Approach for Characterization of Polyphenolic Compounds in *Laminaria japonica*, *Undaria pinnatifida*, *Sargassum fusiforme* and *Ascophyllum nodosum*. Foods.

[18] Bi D., Yang X., Lu J., Xu X. (2022). Preparation and potential applications of alginate oligosaccharides. Crit. Rev. Food Sci. Nutr..

[19] Li S., He N., Wang L. (2019). Efficiently Anti-Obesity Effects of Unsaturated Alginate Oligosaccharides (UAOS) in High-Fat Diet (HFD)-Fed Mice. Mar. Drugs.

[20] Wang M., Chen L., Zhang Z. (2021). Potential applications of alginate oligosaccharides for biomedicine—A mini review. Carbohydr. Polym..

[21] Wu J., Wu M., Zhang H., Zhan X., Wu N. (2021). An Oligomannuronic Acid-Sialic Acid Conjugate Capable of Inhibiting Abeta42 Aggregation and Alleviating the Inflammatory Response of BV-2 Microglia. Int. J. Mol. Sci..

[22] Hao C., Hao J., Wang W., Han Z., Li G., Zhang L., Zhao X., Yu G. (2011). Insulin sensitizing effects of oligomannuronate-chromium (III) complexes in C2C12 skeletal muscle cells. PLoS ONE.

[23] Bi D., Yao L., Lin Z., Chi L., Li H., Xu H., Du X., Liu Q., Hu Z., Lu J. (2021). Unsaturated mannuronate oligosaccharide ameliorates beta-amyloid pathology through autophagy in Alzheimer’s disease cell models. Carbohydr. Polym..

[24] Wen L., Yang H., Ma L., Fu P. (2021). The roles of NLRP3 inflammasome-mediated signaling pathways in hyperuricemic nephropathy. Mol. Cell. Biochem..

[25] Yin H., Liu N., Chen J. (2022). The Role of the Intestine in the Development of Hyperuricemia. Front. Immunol..

[26] Crane J.K. (2013). Role of host xanthine oxidase in infection due to enteropathogenic and Shiga-toxigenic *Escherichia coli*. Gut Microbes.

[27] Han J., Wang Z., Lu C., Zhou J., Li Y., Ming T., Zhang Z., Wang Z.J., Su X. (2021). The gut microbiota mediates the protective effects of anserine supplementation on hyperuricaemia and associated renal inflammation. Food Funct..

[28] Wei J., Zhang Y., Dalbeth N., Terkeltaub R., Yang T., Wang Y., Yang Z., Li J., Wu Z., Zeng C. (2022). Association between Gut Microbiota and Elevated Serum Urate in Two Independent Cohorts. Arthritis Rheumatol..

[29] Xu D., Lv Q., Wang X., Cui X., Zhao P., Yang X., Liu X., Yang W., Yang G., Wang G. (2019). Hyperuricemia is associated with impaired intestinal permeability in mice. Am. J. Physiol. Gastrointest. Liver Physiol..

[30] Li M., Li G., Shang Q., Chen X., Liu W., Pi X., Zhu L., Yin Y., Yu G., Wang X. (2016). In vitro fermentation of alginate and its derivatives by human gut microbiota. Anaerobe.

[31] Zhang N., Liu J., Guo X., Li S., Wang F., Wang M. (2021). Armillaria luteo-virens Sacc Ameliorates Dextran Sulfate Sodium Induced Colitis through Modulation of Gut Microbiota and Microbiota-Related Bile Acids. Nutrients.

[32] Sung Y.Y., Kim D.S. (2021). Eggshell Membrane Ameliorates Hyperuricemia by Increasing Urate Excretion in Potassium Oxonate-Injected Rats. Nutrients.

[33] Li S., Wang L., Liu B., He N. (2020). Unsaturated alginate oligosaccharides attenuated obesity-related metabolic abnormalities by modulating gut microbiota in high-fat-diet mice. Food Funct..

[34] Cao W., Wang C., Chin Y., Chen X., Gao Y., Yuan S., Xue C., Wang Y., Tang Q. (2019). DHA-phospholipids (DHA-PL) and EPA-phospholipids (EPA-PL) prevent intestinal dysfunction induced by chronic stress. Food Funct..

[35] Baltazar-Diaz T.A., Gonzalez-Hernandez L.A., Aldana-Ledesma J.M., Pena-Rodriguez M., Vega-Magana A.N., Zepeda-Morales A.S.M., Lopez-Roa R.I., Del Toro-Arreola S., Martinez-Lopez E., Salazar-Montes A.M. (2022). *Escherichia/Shigella*, SCFAs, and Metabolic Pathways—The Triad That Orchestrates Intestinal Dysbiosis in Patients with Decompensated Alcoholic Cirrhosis from Western Mexico. Microorganisms.

[36] Xu Y., Cao X., Zhao H., Yang E., Wang Y., Cheng N., Cao W. (2021). Impact of Camellia japonica Bee Pollen Polyphenols on Hyperuricemia and Gut Microbiota in Potassium Oxonate-Induced Mice. Nutrients.

[37] Turnbaugh P.J., Ley R.E., Mahowald M.A., Magrini V., Mardis E.R., Gordon J.I. (2006). An obesity-associated gut microbiome with increased capacity for energy harvest. Nature.

[38] Wang M., Zhang S., Zhong R., Wan F., Chen L., Liu L., Yi B., Zhang H. (2021). Olive Fruit Extracts Supplement Improve Antioxidant Capacity via Altering Colonic Microbiota Composition in Mice. Front. Nutr..

[39] Wang J., Chen Y., Zhong H., Chen F., Regenstein J., Hu X., Cai L., Feng F. (2022). The gut microbiota as a target to control hyperuricemia pathogenesis: Potential mechanisms and therapeutic strategies. Crit. Rev. Food Sci. Nutr..

[40] Hao S., Zhang C., Song H. (2016). Natural Products Improving Hyperuricemia with Hepatorenal Dual Effects. Evid.-Based Complement. Altern. Med..

[41] Fan C.Y., Wang M.X., Ge C.X., Wang X., Li J.M., Kong L.D. (2014). Betaine supplementation protects against high-fructose-induced renal injury in rats. J. Nutr. Biochem..

[42] Nistala R., Habibi J., Lastra G., Manrique C., Aroor A.R., Hayden M.R., Garro M., Meuth A., Johnson M., Whaley-Connell A. (2014). Prevention of obesity-induced renal injury in male mice by DPP4 inhibition. Endocrinology.

[43] Liang G., Nie Y., Chang Y., Zeng S., Liang C., Zheng X., Xiao D., Zhan S., Zheng Q. (2019). Protective effects of Rhizoma smilacis glabrae extracts on potassium oxonate- and monosodium urate-induced hyperuricemia and gout in mice. Phytomedicine.

[44] Wang G., Zuo T., Li R. (2020). The mechanism of Arhalofenate in alleviating hyperuricemia-Activating PPARgamma thereby reducing caspase-1 activity. Drug Dev. Res..

[45] Wu Y.L., Chen J.F., Jiang L.Y., Wu X.L., Liu Y.H., Gao C.J., Wu Y., Yi X.Q., Su Z.R., Cai J. (2021). The Extract of Sonneratia apetala Leaves and Branches Ameliorates Hyperuricemia in Mice by Regulating Renal Uric Acid Transporters and Suppressing the Activation of the JAK/STAT Signaling Pathway. Front. Pharmacol..

[46] Mei Y., Dong B., Geng Z., Xu L. (2022). Excess Uric Acid Induces Gouty Nephropathy Through Crystal Formation: A Review of Recent Insights. Front. Endocrinol..

[47] Chen Y., Li C., Duan S., Yuan X., Liang J., Hou S. (2019). Curcumin attenuates potassium oxonate-induced hyperuricemia and kidney inflammation in mice. Biomed. Pharmacother..

[48] Zhang Z.C., Zhou Q., Yang Y., Wang Y., Zhang J.L. (2019). Highly Acylated Anthocyanins from Purple Sweet Potato (*Ipomoea batatas* L.) Alleviate Hyperuricemia and Kidney Inflammation in Hyperuricemic Mice: Possible Attenuation Effects on Allopurinol. J. Agric. Food Chem..

[49] Guo Y., Yu Y., Li H., Ding X., Li X., Jing X., Chen J., Liu G., Lin Y., Jiang C. (2021). Inulin supplementation ameliorates hyperuricemia and modulates gut microbiota in Uox-knockout mice. Eur. J. Nutr..

[50] Ghallab D.S., Shawky E., Metwally A.M., Celik I., Ibrahim R.S., Mohyeldin M.M. (2022). Integrated in silico–in vitro strategy for the discovery of potential xanthine oxidase inhibitors from Egyptian propolis and their synergistic effect with allopurinol and febuxostat. RSC Adv..

[51] Yan F., Xue X., Lu J., Dalbeth N., Qi H., Yu Q., Wang C., Sun M., Cui L., Liu Z. (2022). Superiority of low-dose benzbromarone to low-dose febuxostat in a prospective, randomized comparative effectiveness trial in gout patients with renal uric acid underexcretion. Arthritis Rheumatol..

[52] Keenan R.T. (2020). The biology of urate. Semin. Arthritis Rheum..

[53] Yong T., Liang D., Chen S., Xiao C., Gao X., Wu Q., Xie Y., Huang L., Hu H., Li X. (2022). Caffeic acid phenethyl ester alleviated hypouricemia in hyperuricemic mice through inhibiting XOD and up-regulating OAT3. Phytomedicine.

[54] Le Y., Zhou X., Zheng J., Yu F., Tang Y., Yang Z., Ding G., Chen Y. (2020). Anti-Hyperuricemic Effects of Astaxanthin by Regulating Xanthine Oxidase, Adenosine Deaminase and Urate Transporters in Rats. Mar. Drugs.

[55] Pavelcova K., Bohata J., Pavlikova M., Bubenikova E., Pavelka K., Stiburkova B. (2020). Evaluation of the Influence of Genetic Variants of SLC2A9 (GLUT9) and SLC22A12 (URAT1) on the Development of Hyperuricemia and Gout. J. Clin. Med..

[56] Sun H.L., Wu Y.W., Bian H.G., Yang H., Wang H., Meng X.M., Jin J. (2021). Function of Uric Acid Transporters and Their Inhibitors in Hyperuricaemia. Front. Pharmacol..

[57] Mehmood A., Zhao L., Wang C., Hossen I., Raka R.N., Zhang H. (2019). Stevia residue extract increases intestinal uric acid excretion via interactions with intestinal urate transporters in hyperuricemic mice. Food Funct..

[58] Chau Y.T., Chen H.Y., Lin P.H., Hsia S.M. (2019). Preventive Effects of Fucoidan and Fucoxanthin on Hyperuricemic Rats Induced by Potassium Oxonate. Mar. Drugs.

[59] Wang Z., Li Y., Liao W., Huang J., Liu Y., Li Z., Tang J. (2022). Gut microbiota remodeling: A promising therapeutic strategy to confront hyperuricemia and gout. Front. Cell. Infect. Microbiol..

[60] Pan L., Han P., Ma S., Peng R., Wang C., Kong W., Cong L., Fu J., Zhang Z., Yu H. (2020). Abnormal metabolism of gut microbiota reveals the possible molecular mechanism of nephropathy induced by hyperuricemia. Acta Pharm. Sin. B.

[61] Li X., Chen Y., Gao X., Wu Y., El-Seedi H.R., Cao Y., Zhao C. (2021). Antihyperuricemic Effect of Green Alga Ulva lactuca Ulvan through Regulating Urate Transporters. J. Agric. Food Chem..

[62] Chou Y.T., Kan W.C., Shiao C.C. (2022). Acute Kidney Injury and Gut Dysbiosis: A Narrative Review Focus on Pathophysiology and Treatment. Int. J. Mol. Sci..

[63] Mendez-Salazar E.O., Martinez-Nava G.A. (2022). Uric acid extrarenal excretion: The gut microbiome as an evident yet understated factor in gout development. Rheumatol. Int..

[64] Cao J., Liu Q., Hao H., Bu Y., Tian X., Wang T., Yi H. (2022). Lactobacillus paracasei X11 Ameliorates Hyperuricemia and Modulates Gut Microbiota in Mice. Front. Immunol..

[65] Cao J., Bu Y., Hao H., Liu Q., Wang T., Liu Y., Yi H. (2022). Effect and Potential Mechanism of Lactobacillus plantarum Q7 on Hyperuricemia in vitro and in vivo. Front. Nutr..

[66] Guo W., Xiang Q., Mao B., Tang X., Cui S., Li X., Zhao J., Zhang H., Chen W. (2021). Protective Effects of Microbiome-Derived Inosine on Lipopolysaccharide-Induced Acute Liver Damage and Inflammation in Mice via Mediating the TLR4/NF-kappaB Pathway. J. Agric. Food Chem..

[67] Wu C., Hu Q., Peng X., Luo J., Zhang G. (2022). Marine Fish Protein Peptide Regulating Potassium Oxonate-Induced Intestinal Dysfunction in Hyperuricemia Rats Helps Alleviate Kidney Inflammation. J. Agric. Food Chem..

[68] Zhao X., Jiang L., Fang X., Guo Z., Wang X., Shi B., Meng Q. (2022). Host-microbiota interaction-mediated resistance to inflammatory bowel disease in pigs. Microbiome.

[69] Zheng Q.X., Wang H.W., Jiang X.M., Ge L., Lai Y.T., Jiang X.Y., Huang P.P., Chen F., Chen X.Q. (2022). Changes in the Gut Metabolic Profile of Gestational Diabetes Mellitus Rats Following Probiotic Supplementation. Front. Microbiol..

[70] Kong C., Gao R., Yan X., Huang L., He J., Li H., You J., Qin H. (2019). Alterations in intestinal microbiota of colorectal cancer patients receiving radical surgery combined with adjuvant CapeOx therapy. Sci. China Life Sci..

[71] Gryaznova M., Dvoretskaya Y., Burakova I., Syromyatnikov M., Popov E., Kokina A., Mikhaylov E., Popov V. (2022). Dynamics of Changes in the Gut Microbiota of Healthy Mice Fed with Lactic Acid Bacteria and Bifidobacteria. Microorganisms.

[72] Li L., Ma L., Wen Y., Xie J., Yan L., Ji A., Zeng Y., Tian Y., Sheng J. (2022). Crude Polysaccharide Extracted from Moringa oleifera Leaves Prevents Obesity in Association With Modulating Gut Microbiota in High-Fat Diet-Fed Mice. Front. Nutr..

[73] Grant C.V., Loman B.R., Bailey M.T., Pyter L.M. (2021). Manipulations of the gut microbiome alter chemotherapy-induced inflammation and behavioral side effects in female mice. Brain Behav. Immun..

[74] Liu Y., Li T., Alim A., Ren D., Zhao Y., Yang X. (2019). Regulatory Effects of Stachyose on Colonic and Hepatic Inflammation, Gut Microbiota Dysbiosis, and Peripheral CD4(+) T Cell Distribution Abnormality in High-Fat Diet-Fed Mice. J. Agric. Food Chem..

[75] Zou X.Y., Zhang M., Tu W.J., Zhang Q., Jin M.L., Fang R.D., Jiang S. (2022). Bacillus subtilis inhibits intestinal inflammation and oxidative stress by regulating gut flora and related metabolites in laying hens. Animal.

[76] Liang M., Liu J., Chen W., He Y., Kahaer M., Li R., Tian T., Liu Y., Bai B., Cui Y. (2022). Diagnostic model for predicting hyperuricemia based on alterations of the gut microbiome in individuals with different serum uric acid levels. Front. Endocrinol..

