# Supplementary material for: Ameliorative Effect of Mannuronate Oligosaccharides on Hyperuricemic Mice via Promoting Uric Acid Excretion and Modulating Gut Microbiota
Source: Nutrients. 2023 Jan 13;15(2):417. doi: 10.3390/nu15020417 (PMC9865265; doi:10.3390/nu15020417)
Supplement: Supplementary file 1 [file nutrients-15-00417-s001.zip › nutrients-2056019-supplementary.pdf]

Table S1. The variability of the intestinal flora structure between groups.

combination	SumsOfSqs	MeanSqs	F.Model	R2	P.value	P.value.corrected
M <-> MOS	0.240306	0.240306	2.972353	0.175130	0.000999	0.001998
M <-> N	0.671886	0.671886	7.849656	0.359258	0.001998	0.001998
MOS <-> N	0.970021	0.970021	9.661265	0.408316	0.001998	0.001998

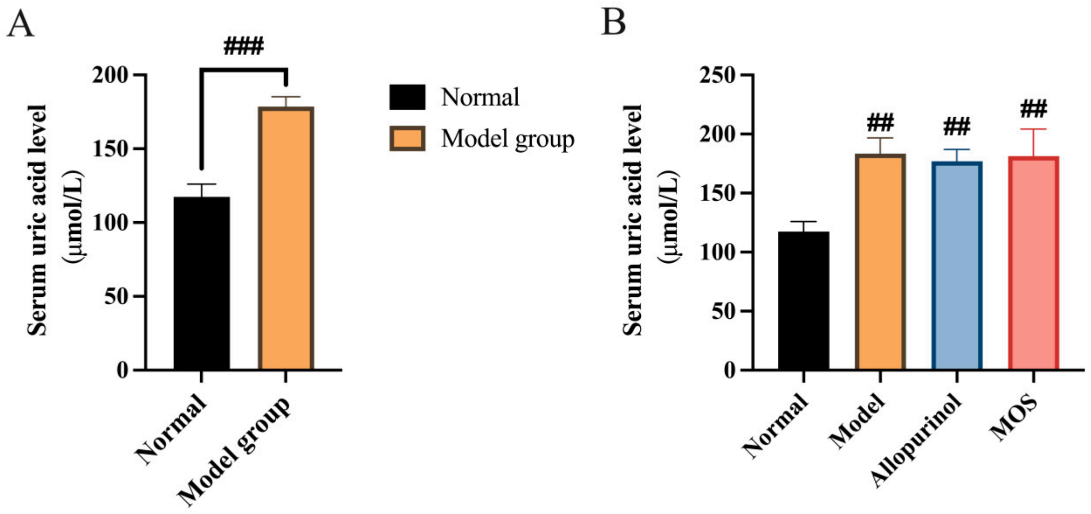

Figure S1. Serum uric acid levels after four weeks of induced hyperuricemia in the first experiments. Normal vs Model group (A); Normal vs Model, Allopurinol, and MOS group (B). ## $p < 0.01$ and ### $p < 0.001$ versus normal.

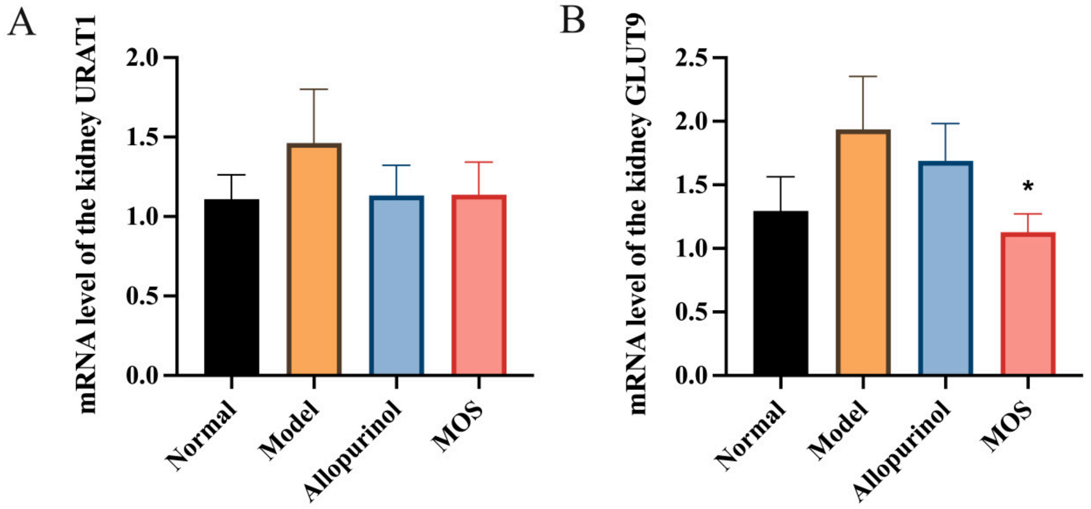

Figure S2. Effect of MOS on mRNA levels of uric acid transporters. mRNA level of the kidney URAT1 (A); mRNA level of the kidney GLUT9 (B). *$p < 0.05$ versus model.

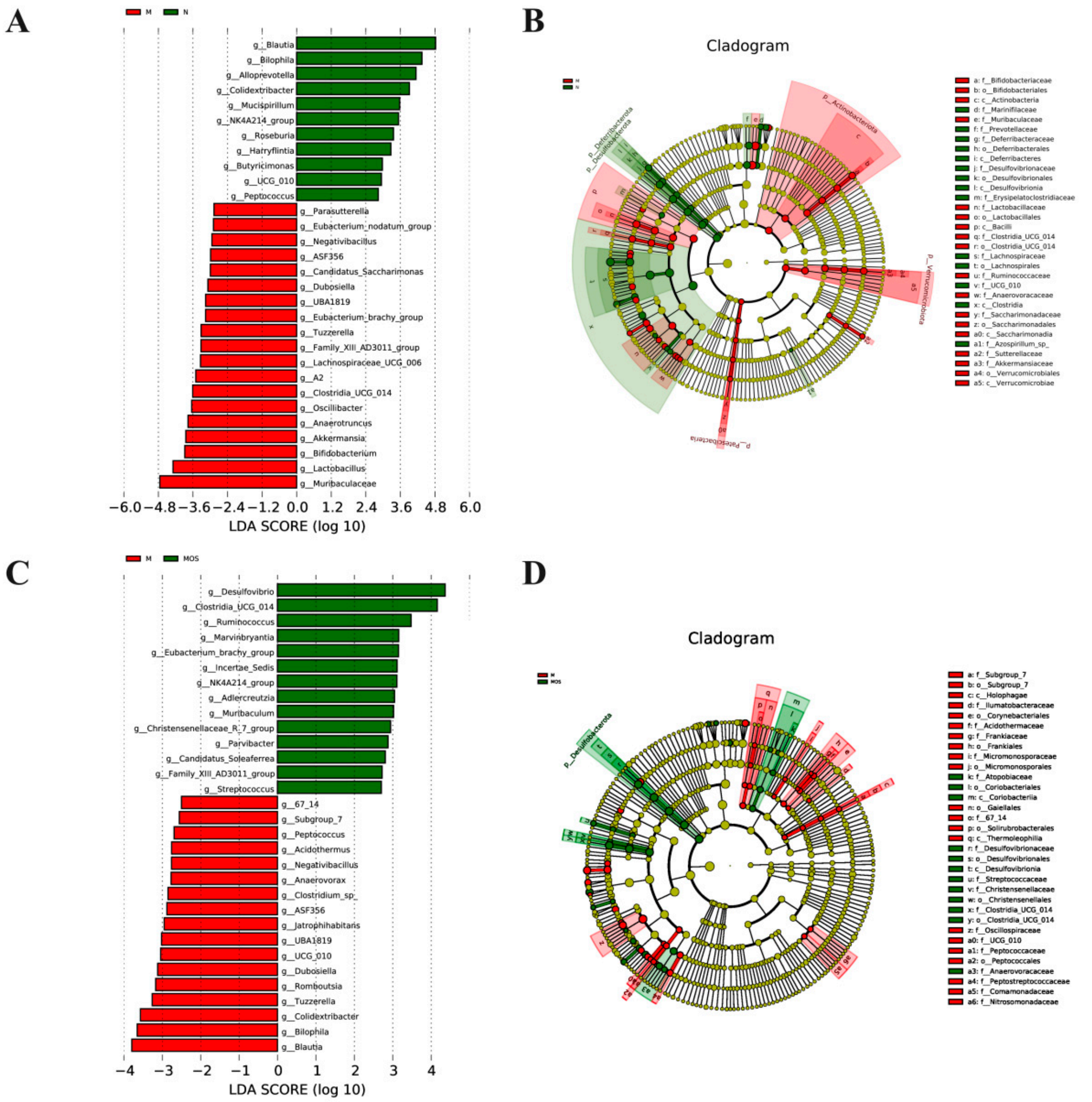

Figure S3. Microbial taxa discrepancies among normal, model, and MOS groups (LDA scores of >2.5 and adjusted p values of <0.05). Histogram of N vs. M (A); Cladogram of N vs. M (B); Histogram of M vs. MOS (C); Cladogram of M vs. MOS (D).

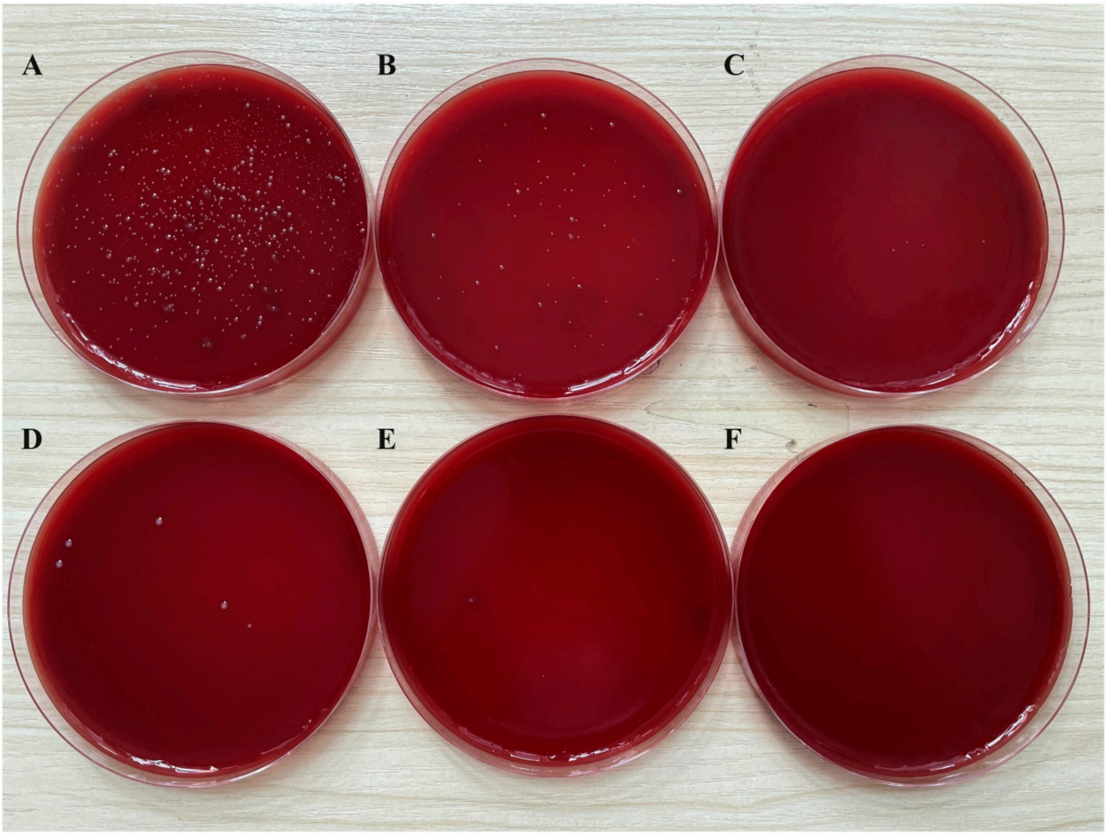

Figure S4. Number of colonies in feces in the second experiments. A to C show the normal group: 10^{-2} dilution gradient (A); 10^{-3} dilution gradient (B); 10^{-4} dilution gradient (C). D to F show the antibiotic group: 10^{-2} dilution gradient (D); 10^{-3} dilution gradient (E); 10^{-4} dilution gradient (F).

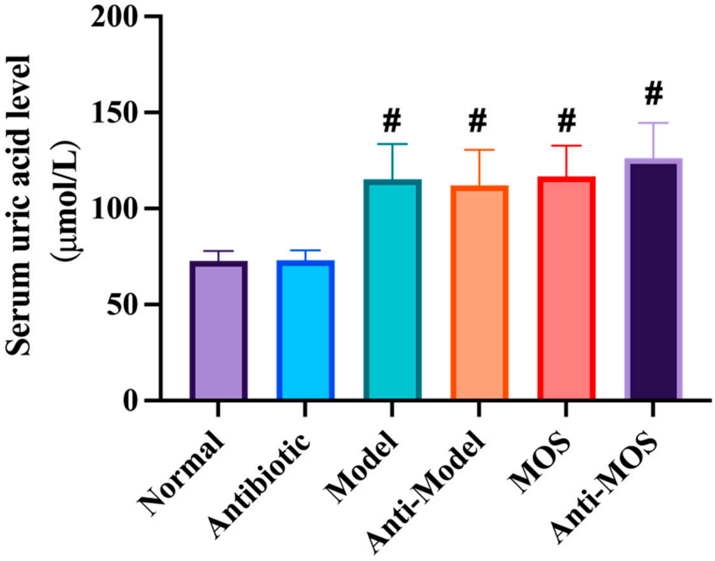

Figure S5. Serum uric acid levels after four weeks of induced hyperuricemia in the second experiments. # $p < 0.05$ versus normal.
